# Human infection with a reassortant swine-origin influenza A(H1N2)v virus in Taiwan, 2021

**DOI:** 10.1186/s12985-022-01794-2

**Published:** 2022-04-07

**Authors:** Ji-Rong Yang, Chuan-Yi Kuo, I.-Ling Yu, Fang-Yen Kung, Fang-Tzy Wu, Jen-Shiou Lin, Ming-Tsan Liu

**Affiliations:** 1 Center for Diagnostics and Vaccine Development, Centers for Disease Control, No. 161, Kun-Yang Street, Taipei, 11561 Taiwan, ROC; 2 Department of Laboratory Medicine, Changhua Christian Hospital, Changhua, Taiwan, ROC

**Keywords:** Swine influenza, Reassortant, Human infection, H1N2v

## Abstract

**Background:**

Influenza A virus infections occur in different species, causing mild-to-severe symptoms that lead to a heavy disease burden. H1N1, H1N2 and H3N2 are major subtypes of swine influenza A viruses in pigs and occasionally infect humans.

**Methods:**

A case infected by novel influenza virus was found through laboratory surveillance system for influenza viruses. Clinical specimens were tested by virus culture and/or real-time RT–PCR. The virus was identified and characterized by gene sequencing and phylogenetic analysis.

**Results:**

In 2021, for the first time in Taiwan, an influenza A(H1N2)v virus was isolated from a 5-year old girl who was suffering from fever, runny nose and cough. The isolated virus was designated A/Taiwan/1/2021(H1N2)v. Full-genome sequencing and phylogenetic analyses revealed that A/Taiwan/1/2021(H1N2)v is a novel reassortant virus containing hemagglutinin (HA) and neuraminidase (NA) gene segments derived from swine influenza A(H1N2) viruses that may have been circulating in Taiwan for decades, and the other 6 internal genes (PB2, PB2, PA, NP, M and NS) are from human A(H1N1)pdm09 viruses.

**Conclusion:**

Notably, the HA and NA genes of A/Taiwan/1/2021(H1N2)v separately belong to specific clades that are unique for Taiwanese swine and were proposed to be introduced from humans in different time periods. Bidirectional transmission between humans and swine contributes to influenza virus diversity and poses the next pandemic threat.

**Supplementary Information:**

The online version contains supplementary material available at 10.1186/s12985-022-01794-2.

## Background

Influenza A viruses infect a variety of host species, including avian, swine, and humans, and cause mild-to-severe symptoms that lead to a heavy disease burden [1]. Influenza A viruses can cause cross-species infection, contributing to the generation of novel virus variants that have resulted in global pandemics, such as influenza A(H1N1) in 1918, A(H2N2) in 1957, A(H3N2) in 1968 and A(H1N1)pdm09 in 2009 [2, 3]. Generally, swine are considered to serve as a ‘mixing-vessel’ host for the generation of pandemic viruses because they can be infected by both avian and human influenza A viruses [4]. The 2009 influenza pandemic demonstrated this hypothesis since it was caused by a swine-origin influenza A(H1N1) virus with a unique reassortment of recent North American H3N2 and H1N2 swine viruses (avian/human/swine ‘triple’ reassortant viruses) with Eurasian avian-like swine viruses. After this quadruple reassortment, the virus contained PB2 and PA genes of North American avian virus origin, a PB1 gene of human H3N2 virus origin, HA, NP, and NS genes of classical swine virus origin, and NA and M genes of Eurasian avian-like swine virus origin [2, 5]. However, repeated spillovers of pandemic viruses from humans to swine have occurred, leading to substantial virus evolution and seeding swine globally with new viral diversity [6]. In the USA, influenza A(H1N1) virus was first isolated from swine in 1930 and was considered to derive from the human pandemic virus of 1918 [7, 8]. Thereafter, with multiple introductions of human and avian influenza A viruses into the swine population in different time periods, multiple genotypes and clades of A(H1) and A(H3) viruses have been generated through inter- and intra-subtype reassortment [9]. Later, upon the 2009 pandemic, the viral gene pools of human influenza A(H1N1)pdm09 viruses were reintroduced into the swine population, resulting in the circulation of different genotypes in swine and increasing the threat of the next pandemic [10–14]. Human infections caused by influenza A(H1)v and A(H3)v viruses with the internal protein gene(s) from A(H1N1)pdm09 have been sporadically reported [15–18]. In this study, we reported a human case infected by a reassortant swine influenza A(H1N2)v virus that harbors HA and NA gene segments derived from swine influenza A(H1N2) in Taiwan and 6 internal genes from human A(H1N1)pdm09 viruses. We present a description of the long-term establishment of swine influenza viruses containing a unique HA gene (from classical swine A(H1N1)) and a human-origin NA gene (from A(H3N2), 1970s) in Taiwan. We also characterized and compared the viral full-genome sequences of isolates from humans infected by swine influenza A(H1N1) and A(H1N2), highlighting the increase in geographical differences regarding genetic diversity in swine influenza after the 2009 pandemic.

## Methods

### Collection of clinical specimens and virus isolates

In Taiwan, there is a well-established laboratory surveillance system for influenza viruses circulating in the community. Clinical specimens are routinely collected from outpatients with influenza-like illnesses and transported to commissioned laboratories for influenza testing by virus culture and/or real-time RT-PCR [19]. All the influenza isolates obtained from positive cases are transported to the Taiwan Centers for Disease Control (CDC) for further virological characterization. The surveillance system is coordinated by the Taiwan CDC and serves as the basis for a prompt response to yearly seasonal influenza epidemics or unexpected pandemics caused by a novel virus strain.

### Influenza viral gene sequencing and phylogenetic analysis

To investigate the molecular phylogenies of the influenza A(H1N2)v virus identified in this study, full-genome sequences of the previously identified representative influenza A(H1)v viruses during the period from 2015 to 2021 in the USA ((H1N1)v and (H1N2)v), Germany ((H1N1)v), the Netherlands ((H1N1)v), Denmark ((H1N1)v), Canada ((H1N2)v) and Brazil ((H1N2)v) as well as human seasonal influenza A(H1N1), A(H1N1)pdm09 and A(H3N2) viruses isolated between 1968 and 2019, several swine and avian influenza A viruses were selected as reference sequences and included in detailed analyses (Additional file 1: Table S1). Viral RNA extraction and nucleotide sequence analysis of viral genes were performed as previously described [20]. Multiple sequence alignments, protein translation and phylogenetic analysis were performed on the basis of nucleotide sequences using MEGA6 software [21] and BioEdit (http://www.mbio.ncsu.edu/BioEdit/bioedit.html). Phylogenetic trees were constructed using the neighbor-joining method, and 1000 bootstrap replications were performed to evaluate the robustness.

### Sequence information

The full-length genomic sequences of the A/Taiwan/1/2021(H1N2)v virus were analyzed to investigate the phylogenetic and genetic characteristics of this virus (GISAID accession numbers EPI1918923-EPI1918930).

## Results

### The patient and epidemiology survey

The influenza A(H1N2)v virus was isolated from a 5-year-old child who lives in the county of central Taiwan through the nationwide laboratory surveillance network. She developed symptoms of cough and fever on March 12 and 13, 2021, respectively, and visited the emergency department of a local hospital on March 14. Throat swab specimens were collected at initial admission, tested positive for influenza A by rapid immunochromatography and were further subjected to influenza virus isolation. Oseltamivir was given empirically on the day of her admission without hospitalization. A total of 6 family members, including her great-grandmother, grandfather, grandmother, father, mother and younger brother, were notified, four of whom (grandfather, grandmother, father and mother) were pig farm workers. Notably, the father, mother and younger brother had exhibited mild respiratory symptoms (cough or runny nose) during March 15–22; however, no respiratory specimens had been collected or tested for influenza. The case patient and her younger brother sometimes went to the pig farm on holidays with their family, they did not directly contact with pigs based on the epidemiological investigation.

### Virus isolation and phylogenetic analyses of the influenza A(H1N2)v virus

The virus was successfully propagated in MDCK cells. The initial identification of the virus by one local laboratory of the network influenza surveillance using real-time RT–PCR revealed an unsubtyped influenza A virus since only a matrix protein (M) gene assay of influenza A was positive, whereas subtyping assays targeting HA genes of human A(H1)pdm09 and A(H3) viruses were both negative. The virus isolate was then sent to the Taiwan CDC and further characterized as a subtype of A(H1N2)v on the basis of the full-length sequences of the viral HA and NA genes (designated A/Taiwan/1/2021(H1N2)v, TW01/21 in short). Furthermore, throat swab specimens were also obtained from all 6 family members on April 8, 2021, and all tested negative for influenza virus with real-time RT–PCR assays by the Taiwan CDC.

**Fig. 1:**
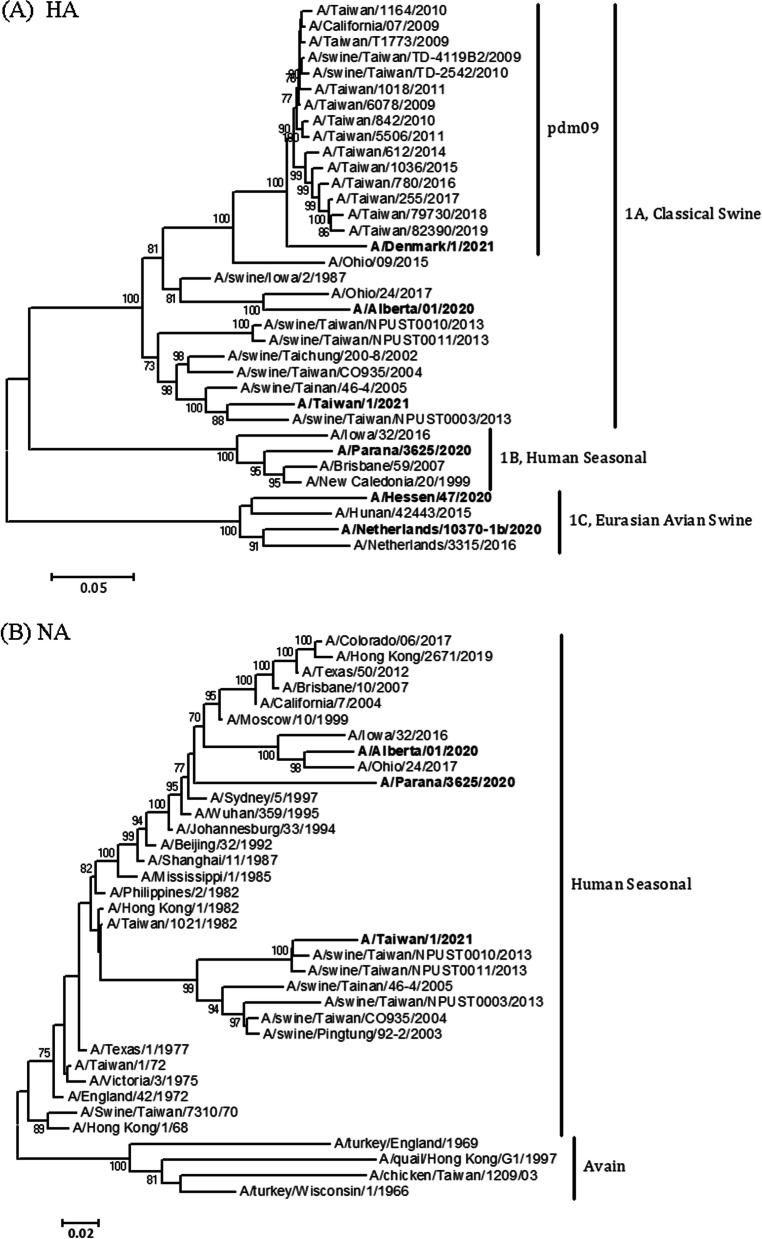
Phylogenetic relationships of surface protein-encoding genes for **A** HA and **B** NA of the A/Taiwan/1/2021(H1N2)v virus. Classification of the specific evolutionary clades is indicated. The recently identified influenza A(H1)v viruses in Canada, Denmark, Brazil, Germany and the Netherlands are included as reference sequences shown in bold. Branch values of more than 70 are indicated

The full-length complete genomic sequences of the TW01/21 virus were successfully determined. Regarding the evolutionary relationship of TW01/21 with other globally identified influenza A(H1)v viruses, phylogenetic analyses of the eight viral gene segments were conducted. Based on the phylogeny of viral HA gene sequences (Fig. 1A), TW01/21 was designated clade 1A (classical swine-like), with the nomenclature aligning with that of the World Health Organization (WHO) [20]. TW01/21 was phylogenetically separated from the recently identified influenza A(H1)v viruses in the same clade from Canada (A/Alberta/01/2020(H1N2)v, 1A.1, classical swine-like), Denmark (A/Denmark/1/2021(H1N1)v, 1A.3, classical swine-like, H1pdm09 lineage), Brazil (A/Parana/3625/2020(H1N2)v, 1B, human seasonal-like), Germany (A/Hessen/47/2020(H1N1)v, 1C.2, Eurasian avian swine-like) and the Netherlands (A/Netherlands/10370-1b/2020(H1N1)v, 1C.2, Eurasian avian swine-like). Moreover, the HA of TW01/21 indicates that the virus belongs to a specific subclade comprising exclusively a variety of swine influenza H1 viruses isolated during the period from 2002 to 2013 in Taiwan and clusters most closely with those identified in 2013. The NA gene of TW01/21 is human seasonal-like and clusters with Taiwanese swine influenza A(H1N2) viruses, forming a unique subclade apart from the Canadian virus A/Alberta/1/2020 (H1N2)v, whose NA is more phylogenetically similar to those of the modern A(H3N2) viruses circulating during the 2000s and 2010s (Fig. 1B). The remaining 6 internal protein genes (PB2, PB1, PA, NP, M and NS) all were suggested location in clades together with human influenza A(H1N1) viruses of the H1N1pdm09 lineage (Additional file 2: Fig. S1), indicating that the TW01/21 virus is a 2 + 6 reassortant virus.

### Molecular characteristics of the TW01/21 virus

Molecular signatures associated with host adaptation, receptor specificity, pathogenesis and antiviral resistance were also investigated (Table 1). The virus possesses the markers 190D and 225D (H3 numbering) in the HA protein, suggesting preferential binding to the human-type influenza receptor. Substitutions in the NA protein, including E119V, I222L, R224K and R292K, which compromise sensitivity to neuraminidase inhibitors, were not detected. However, the S31N substitution in the M2 protein was identified, suggesting resistance to M2 ion channel inhibitors. Furthermore, the HA cleavage site of the virus comprises no polybasic amino acid residues (PSIQSR/GLF), indicating that the virus belongs to a low-pathogenicity type. The L336M substitution in the polymerase acidic (PA) protein, which is related to increased polymerase activity in mice, was also detected.

**Table 1:** Molecular analysis of the A/Taiwan/1/2021(H1N2)v virus

Determination of viral characteristics					
Protein	Position	Mutations		A/Taiwan/1/2021	Function
PB2	627	E	K	E	Replication ability [26]
	701	D	N	D	Nuclear Import [26]
PB1	99	H	Y	H	Increased transmission in ferret [27]
PA	336	L	M	**M**	Increased polymerase activity in mice [28]
HA (H3 numbering)	Cleavage site	Single basic aa	Multiple basic aa	PSIQSR/GLF	high pathogenesis in poultry [29]
	190	E	D	**D**	Increased binding to human-type influenza receptor [30, 31]
	225	G	D	**D**	Increased binding to human-type influenza receptor [30, 31]
	226	Q	L	Q	Increased binding to human-type influenza receptor [30, 32]
	228	G	**S**	G	Increased binding to human-type influenza receptor [30, 32, 33]
NA (N2 numbering)	119	E	V	E	Resistance to oseltamivir and zanamivir [34]
	222	I	L	I	Resistance to oseltamivir and zanamivir [35]
	224	R	K	R	Resistance to oseltamivir and zanamivir [36]
	292	R	K	R	Resistance to oseltamivir and zanamivir [34]
M2	31	S	N	**N**	Amantadine resistance [37]
NS1	42	P	S	A	Increased pathogenesis in mice [38]
	92	D	E	D	Altered virulence in mice [39]
	205	N	S	N	Altered antiviral response in host [40]

The genetic constellation of TW01/21 and other A(H1)v viruses of 2020–2021 based on the complete genome phylogenies is summarized in Table 2. Accordingly, these A(H1N1)pdm09-like internal protein genes were also detected in A(H1)v viruses of Canada, Denmark and Brazil belonging to different HA clades, suggesting the involvement of intra- and interclade reassortment with human A(H1N1)pdm09 viruses between swine influenza A viruses (Table 2). Furthermore, the internal protein genes of TW01/21 are phylogenetically separated from those of the Taiwanese swine influenza A(H1N2) viruses identified before 2007 (classical swine like), indicating the introduction of human A(H1N1)pdm09 viruses into the swine population in Taiwan after the 2009 pandemic.

**Table 2:** Genetic constellation of the A/Taiwan/1/2021(H1N2)v virus

Viruses	Subtype	Phylogenetic clade (lineage)*							
		PB2	PB1	PA	HA	NP	NA	MP	NS
A/Taiwan/1/2021	(H1N2)v	pdm09	pdm09	pdm09	CS	pdm09	HS	pdm09	pdm09
A/Alberta/01/2020	(H1N2)v	pdm09	pdm09	pdm09	CS	pdm09	HS	pdm09	CS
A/Denmark/1/2021	(H1N1)v	pdm09	pdm09	pdm09	pdm09	pdm09	ND	pdm09	EA
A/Parana/3625/2020	(H1N2)v	pdm09	pdm09	pdm09	HS	pdm09	HS	pdm09	pdm09
A/Hessen/47/2020	(H1N1)v	EA	EA	EA	EA	EA	ND	EA	EA
A/Netherlands/10370-1b/2020	(H1N1)v	EA	EA	EA	EA	EA	ND	EA	EA

^*^EA: Eurasian Avian-like; CS: Classical Swine; HS: Human Seasonal; ND: not determined because of N1 subtype

^*^Phylogenetic clade or lineage is determined based on the respective phylogeny analyzed from full-length complete genomic sequences of each virus

## Discussion

Because of geographical segregation of pig populations and multiple introductions of viruses from either humans or birds, swine influenza viruses differ genetically between continents and regions [22]. In Taiwan, before the 2009 influenza A(H1N1)pdm09 pandemic, HA genes of the domestically circulating swine influenza H1 viruses belonged to 1A.1 (classical swine lineage) [22], which was considered to have originated from the human A(H1N1) pandemic of 1918 [7]. During the 1968 human influenza A(H3N2) pandemic, the human A(H3N2) virus was transmitted to swine and detected in a Taiwan slaughterhouse in 1969 [23]. It was thought that the human-like N2 gene of swine influenza was introduced into the Taiwanese swine population at that time or afterward. After the 2009 pandemic, gene segments of the human A(H1N1)pdm09 virus were reintroduced into endemic swine viruses, resulting in the generation of the TW01/21 virus. Regarding the ancestral origin of the viral genes, TW01/21 was proposed to have acquired different gene segments from three respective human pandemics: the HA gene from 1918 A(H1N1), the NA gene from 1968 A(H3N2) and 6 internal genes from 2009 A(H1N1)pdm09 viruses. Notably, the HA and NA genes in the phylogenetic tree were shown to form a unique cluster with those of swine influenza in Taiwan (Fig. 1), indicating that the HA and NA genes evolved in a separate and isolated manner. Because of different evolutionary and epidemiological characteristics between human and swine influenza viruses, some viral genes that were introduced from human viruses persisted and “frozen” in the swine population for decades. Then, these viruses re-infect susceptible humans of the new generation and potentially cause a new human pandemic. In this study, the N2 genes of Taiwanese swine A(H1N2)/A(H3N2) and TW01/21 viruses were close to those of human A(H3N2) from the 1980s (Fig. 1), which were different from the N2 genes of swine viruses (e.g., A/Alberta/01/2020) in North America that were more closely related to human A(H3N2) in 1990s (Fig. 1), suggesting that the N2 genes of swine influenza A viruses in different regions were introduced from human influenza A viruses in different time periods. After the 2009 pandemic, the gene segments of A(H1N1)pdm09 were reintroduced into the swine population, resulting in the circulation of different genotypes in the swine population [10–14]. Humans infected by reassortant swine influenza viruses with the gene pool of A(H1N1)pdm09 virus have been sporadically detected [15, 16, 18, 24], and it has been reported that Eurasian avian-like swine influenza A(H1N1) virus with A(H1N1)pdm09 genes has facilitated human infection [17]. Although swine influenza viruses isolated from human patients are currently similar in those known to be enzootic in swine populations in the respective regions or countries [25], dynamic and high genetic diversity among these human-isolated swine influenza strains raises the possibility of transmission among humans. The complex interfaces of human and swine viruses contribute to viral diversity and pose the threat of an influenza pandemic. It is important to strengthen the integration and cooperation between the surveillance of animal and human influenza viruses. Our study has several limitations. First, despite the highest similarity of HA (91.5%) and NA (95.5%) genes of the TW01/21 with A/swine/Taiwan/NPUST0003/2013(H1N2) and A/swine/Taiwan/NPUST0011/2013 (H1N2) viruses, respectively, we could not identify the contemporary swine influenza A viruses that may be related to the current infection due to lack of enough sequence information on Taiwanese swine influenza viruses available from open sources. Second, household transmission among the index case and her family members could not be determined since their acute-phase sera were not available. However, although the transmission origin of the index case was unclear due to lack of evidence that she had direct contact with pigs in the pig farm, our data suggest the possible pig-to-human transmission route of this infection.

## Conclusions

In this study, we identified a human case infected by a novel reassortant virus containing HA and NA gene segments derived from swine influenza A(H1N2) viruses that may have been circulating in Taiwan for decades, and the other 6 internal genes (PB2, PB2, PA, NP, M and NS) are from human A(H1N1)pdm09 viruses. The phylogenetic analysis revealed that HA and NA genes separately belong to specific clades that are unique for Taiwanese swine and were proposed to be introduced from humans in different time periods. Bidirectional transmission between humans and swine contributes to influenza virus diversity and poses the next pandemic threat.

## Supplementary Information

**Additional file 1**. **Supplementary Table 1.** The names and accession numbers of viruses were analyzed in this study.

**Additional file 2**. Supplementary Fig. 1. Phylogenetic relationships of representative internal protein-encoding genes for (A) PB2, (B) PB1, (C) PA, (D) NP, (E)M and (F)NS of the A/Taiwan/1/2021(H1N2)v virus. Classification of the specific evolutionary clades is indicated. The recently identified influenza A(H1)v viruses in Canada, Denmark, Brazil, Germany and the Netherlands are included as reference sequences shown in bold. Branch values of more than 70 are indicated.

## Data Availability

All data generated or analyzed during this study are included in this published article.
