# Supplementary figures and images for: Human infection with a reassortant swine-origin influenza A(H1N2)v virus in Taiwan, 2021
Source: Virol J. 2022 Apr 7;19:63. doi: 10.1186/s12985-022-01794-2 (PMC8988477; doi:10.1186/s12985-022-01794-2)

# (A) PB2

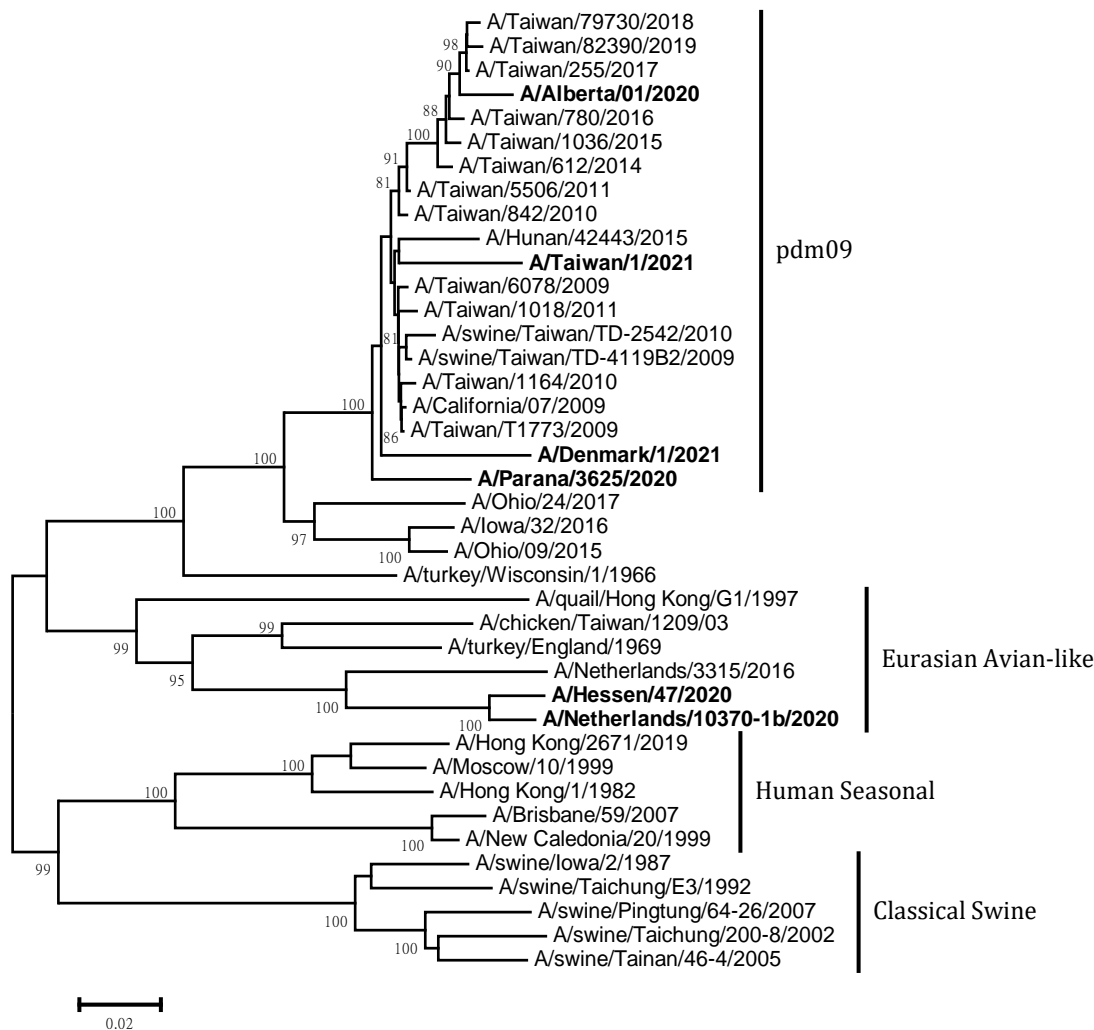

# (B) PB1

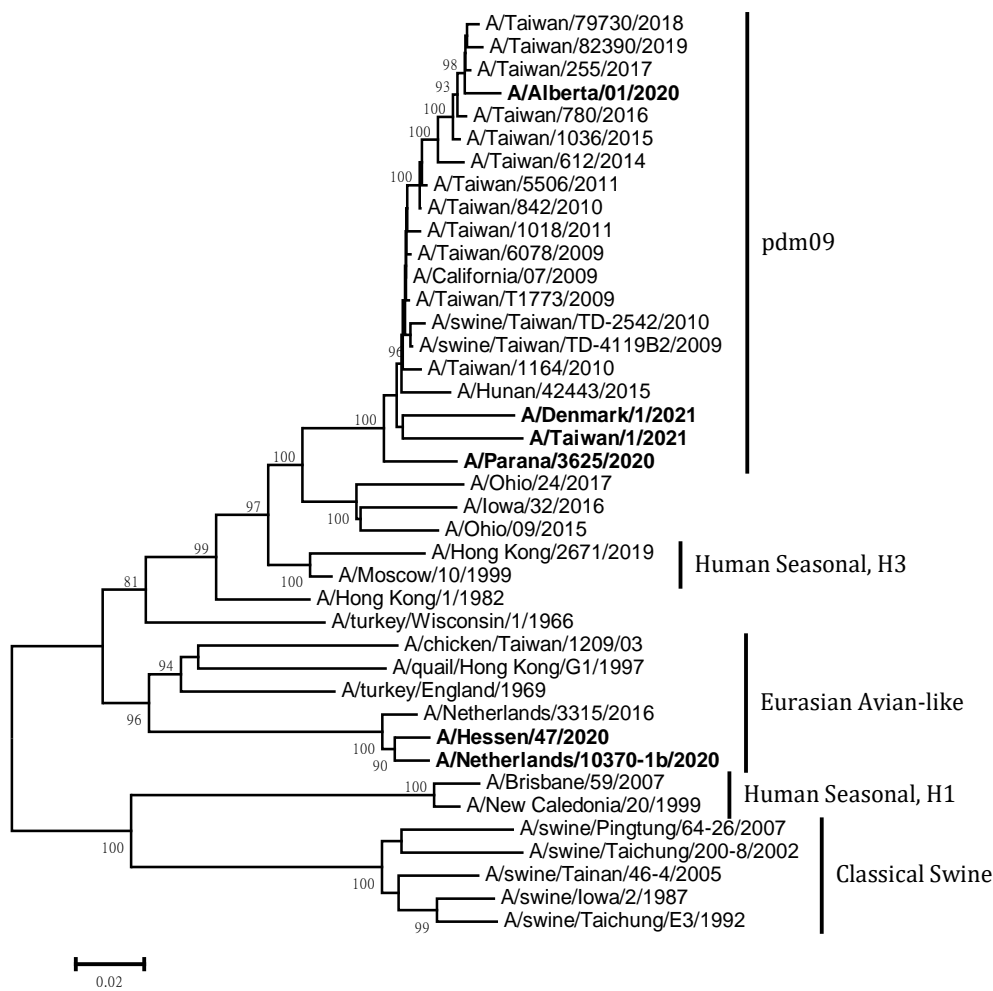

(C) PA

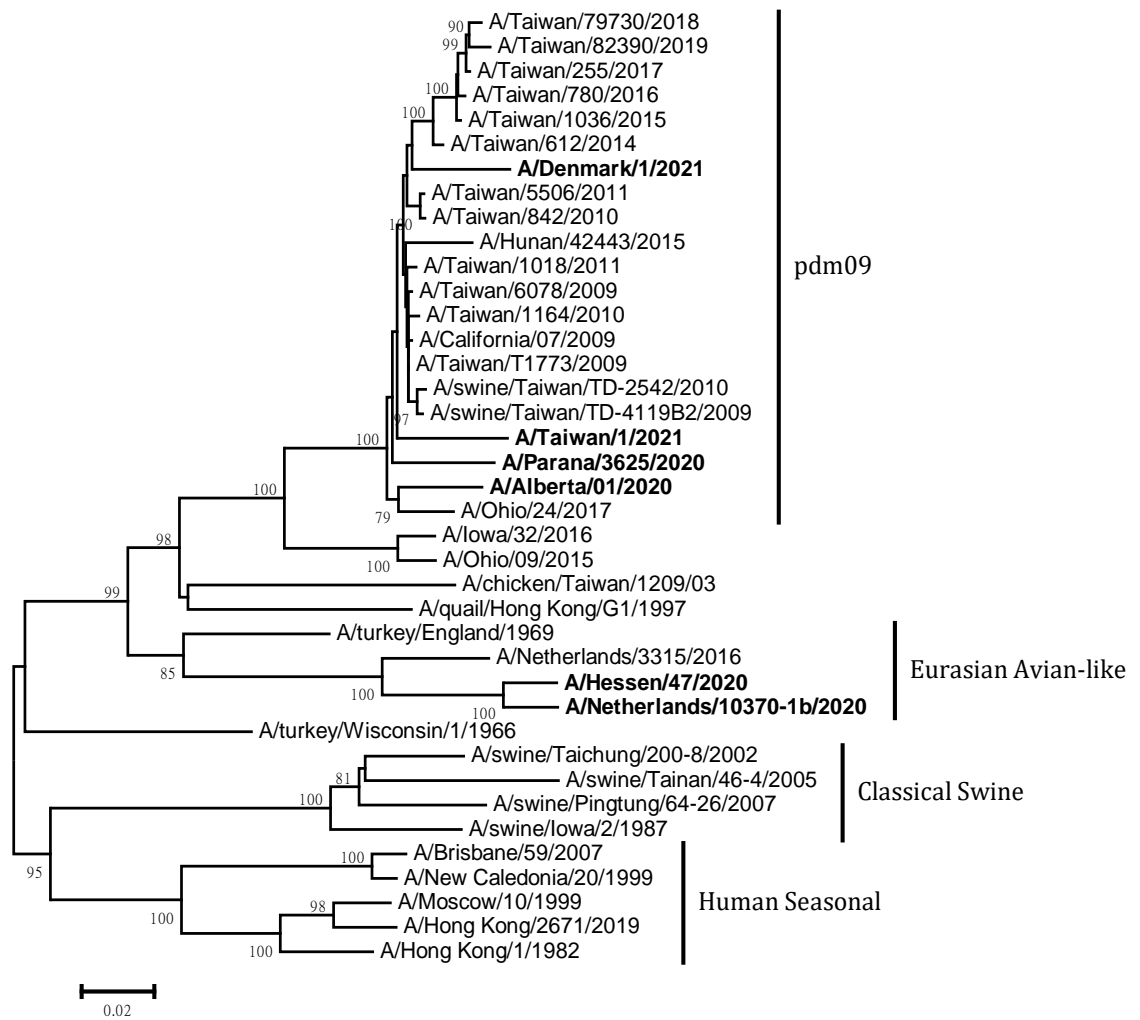

(D) NP

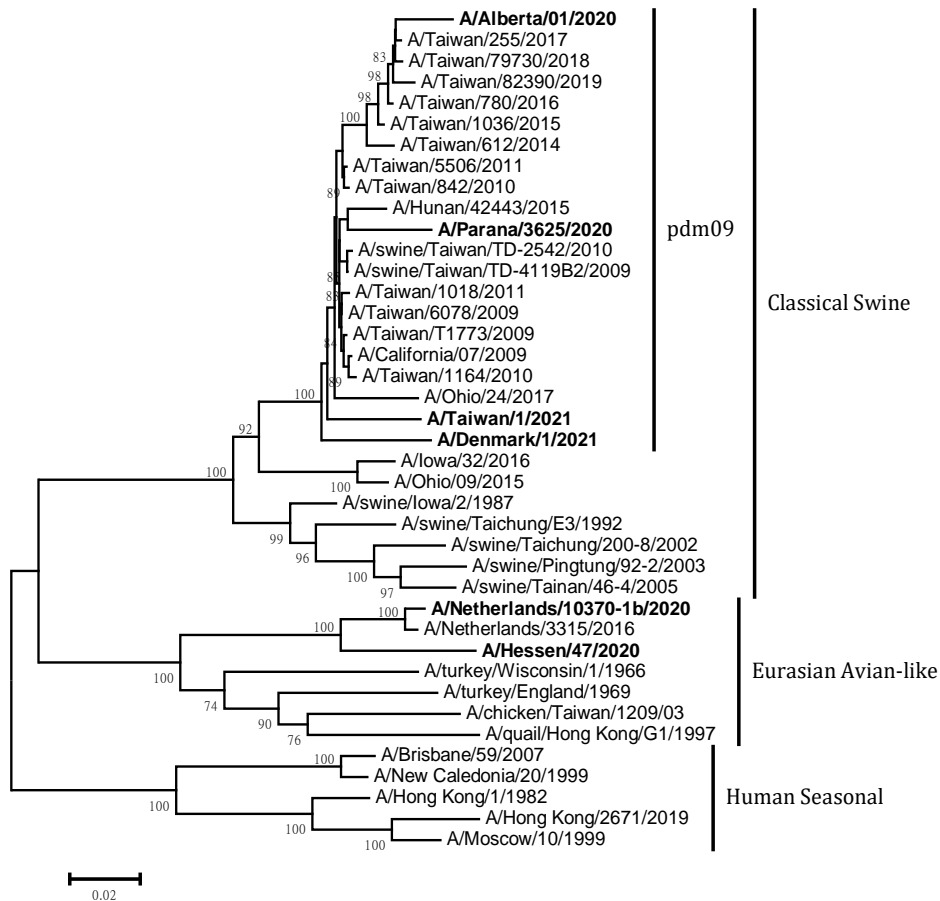

(E) M

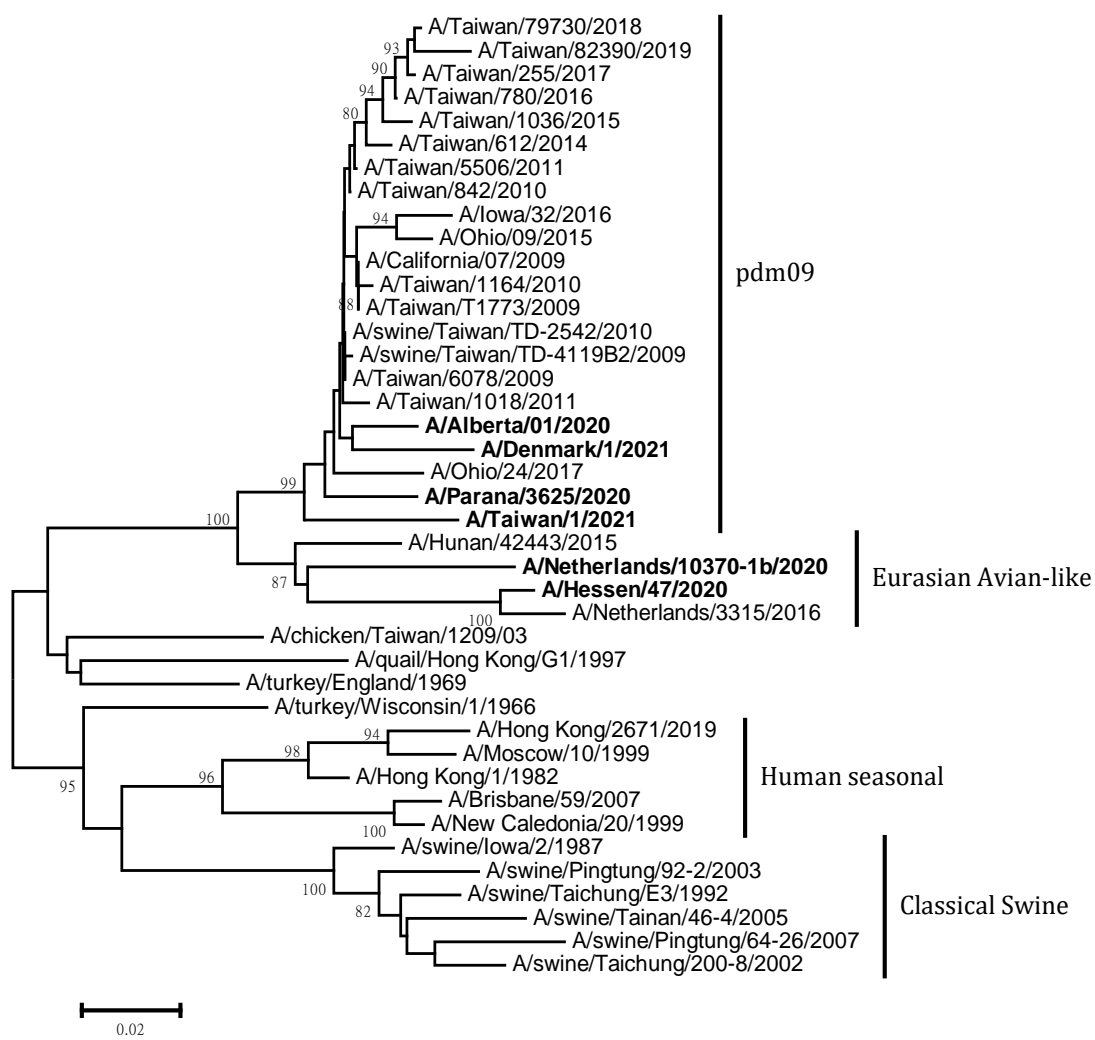

(F) NS

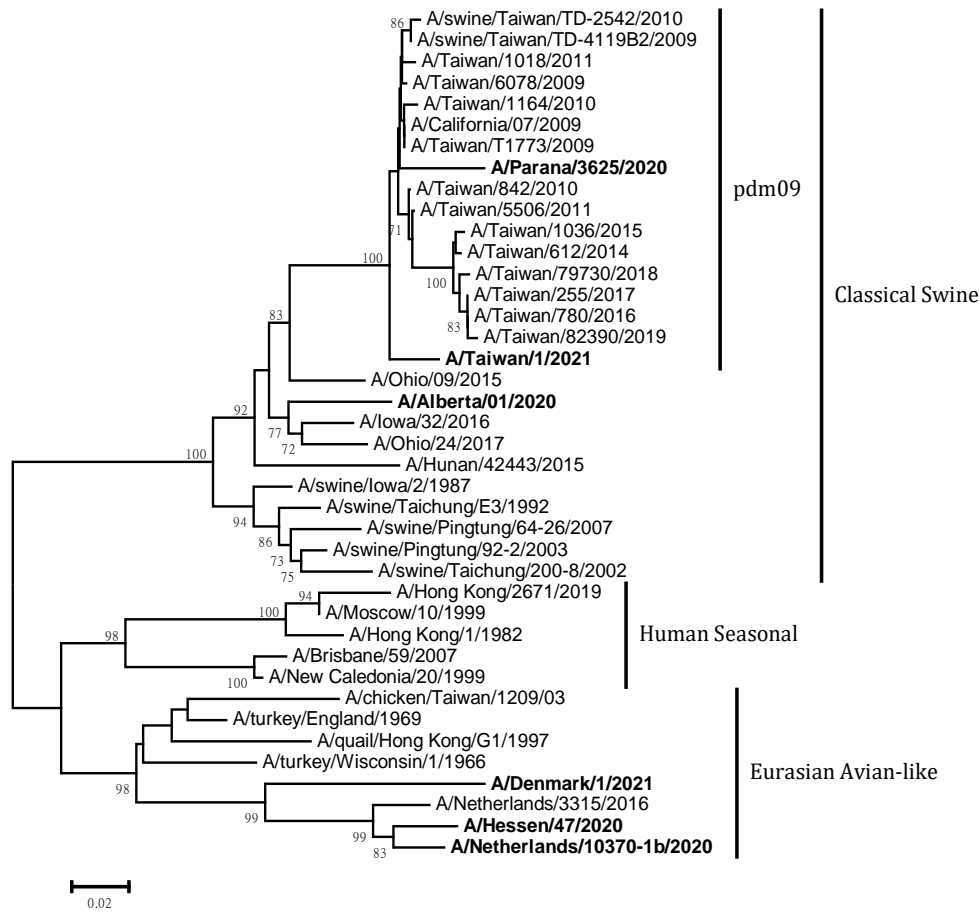

Supplement: Supplementary file 2 — Additional file 2. Supplementary Fig. 1. Phylogenetic relationships of representative internal protein-encoding genes for (A) PB2, (B) PB1, (C) PA, (D) NP, (E)M and (F)NS of the A/Taiwan/1/2021(H1N2)v virus. Classification of the specific evolutionary clades is indicated. The recently identified influenza A(H1)v viruses in Canada, Denmark, Brazil, Germany and the Netherlands are included as reference sequences shown in bold. Branch values of more than 70 are indicated. [file 12985_2022_1794_MOESM2_ESM.pdf]
